# Music notation: a new method for visualizing social interaction in animals and humans

**DOI:** 10.1186/1742-9994-3-18

**Published:** 2006-11-17

**Authors:** Ivan D Chase

**Affiliations:** 1 Department of Sociology, Stony Brook University, Stony Brook, NY 11794-4345, USA; 2 Graduate Program in Ecology and Evolution, Stony Brook University, Stony Brook, NY 11794-5245, USA

## Abstract

**Background:**

Researchers have developed a variety of techniques for the visual presentation of quantitative data. These techniques can help to reveal trends and regularities that would be difficult to see if the data were left in raw form. Such techniques can be of great help in exploratory data analysis, making apparent the organization of data sets, developing new hypotheses, and in selecting effects to be tested by statistical analysis. Researchers studying social interaction in groups of animals and humans, however, have few tools to present their raw data visually, and it can be especially difficult to perceive patterns in these data. In this paper I introduce a new graphical method for the visual display of interaction records in human and animal groups, and I illustrate this method using data taken on chickens forming dominance hierarchies.

**Results:**

This new method presents data in a way that can help researchers immediately to see patterns and connections in long, detailed records of interaction. I show a variety of ways in which this new technique can be used: (1) to explore trends in the formation of both group social structures and individual relationships; (2) to compare interaction records across groups of real animals and between real animals and computer-simulated animal interactions; (3) to search for and discover new types of small-scale interaction sequences; and (4) to examine how interaction patterns in larger groups might emerge from those in component subgroups. In addition, I discuss how this method can be modified and extended for visualizing a variety of different kinds of social interaction in both humans and animals.

**Conclusion:**

This method can help researchers develop new insights into the structure and organization of social interaction. Such insights can make it easier for researchers to explain behavioural processes, to select aspects of data for statistical analysis, to design further studies, and to formulate appropriate mathematical models and computer simulations.

## Background

Considering several examples of social interaction is perhaps the easiest way to explain how the new method introduced here can be applied. These examples could include, say, animals forming a dominance hierarchy, a husband and wife having a discussion, and two primates alternately grooming each other. In some cases, such as the formation of the dominance hierarchy, the interaction will progress to some expected result. The animals will, for example, form a linear hierarchy structure. These kinds of interactions generate questions about the behavioural processes leading to that expected result: Did the animals establish relationships within the hierarchy quickly or were there protracted battles between the pairs? Were there typical sequences of interaction such as losers of encounters being attacked by other group members? Were the interaction records across several groups similar in form? Cases such as the discussion between the husband and wife or the grooming bout in the primates, where there is no clear, overall outcome, can generate questions about the organization of the interaction itself. For example, what kinds of verbal tactics did the husband and wife typically use? Were certain types of responses – questions, statements, criticisms, etc. – typically followed by certain other types? For the primates, did they alternate acts of grooming and, if so, were the acts of approximately the same length, or did one primate groom the other for longer periods of time?

Although we are already making progress in answering such questions, I suggest here that we could increase our understanding by using techniques that allow us to visualize processes of interaction graphically. I introduce some examples of such a new graphical technique here, and I illustrate them using data records from small groups of chickens forming dominance hierarchies.

To get a concrete idea of how this new visualization technique might aid the investigation of social interaction, consider the related case of a researcher interested in the possible relationship between two quantitative variables. Here, one of the first things a researcher routinely does is to look at a scatter plot of the data points. Doing so helps the researcher determine whether there is a relationship between the variables, and if so, whether it is linear or curvilinear, and if curvilinear, its particular shape. After doing this, the researcher is in a much better position to choose appropriate means of analysis. Looking at the scatter plot can also suggest how the researcher might transform the data – by taking logarithms or square roots, for example – to show the relationship between the variables. While features such as liner and curvilinear relationships, outliers, and the need for data transformations are very easy to see in a scatter plot, it would be much more difficult to ascertain such information by simply inspecting the listing of the raw data points in the original data set. In short, viewing the scatter plot allows us to take advantage of our considerable abilities for visual pattern recognition to make readily apparent what was previously obscure in the original, raw data record.

Data records of social interaction present the same problems with respect to seeing patterns, highlighting the need for graphical aids. Interaction data sets can be very large: individuals in small groups of animals or humans may interact with one another hundreds or even thousands of times within a short period. In interaction data sets for groups of animals or humans, a researcher has to track relationships, not just between two variables, as in the regression example above, but between all the possible pairs of individuals in a group. Even in a group as small as four, for example, this amounts to six pairs of individuals. Further, while we already have some well-developed ideas about the kinds of possible relationships between two quantitative variables – linear, curvilinear with S-shaped and U-shaped variations, etc. – we know less about the structure of processes of social interaction, especially those leading to patterns of social organization in groups [1,2].

The upshot of these difficulties is that we need a graphical method allowing us to see social interaction directly, as we can observe data points in a scatter plot directly. Such a method would aid us in thinking and reasoning about patterns in interaction, in developing hypotheses for further testing, and in choosing proper methods of statistical analysis. In addition, if this method allowed us to scan easily back and forth over a whole data set, it would be an even more powerful tool for our perception of regularities, or their lack, in records of social interaction. The method I describe below is designed to meet these requirements. I show how this technique can be used to: (1) display long and detailed records of social interaction in an easily seen and understood form; (2) compare interaction records in different groups of real animals or interaction records in a group of real animals versus those produced by computer simulations attempting to model the same kind of interaction; (3) scan for the occurrence and context of various kinds of small-scale sequences of interaction in groups; and (4) show how the overall record of interaction in a group can be broken down to reveal the various patterns of interaction in subgroups. In the Discussion, I consider modifications and extensions of the basic techniques that might be needed to show social interaction data in a variety of situations beyond those of the hens forming dominance hierarchies.

## Results

### Visualization of interaction records of groups

**Figure 1:**
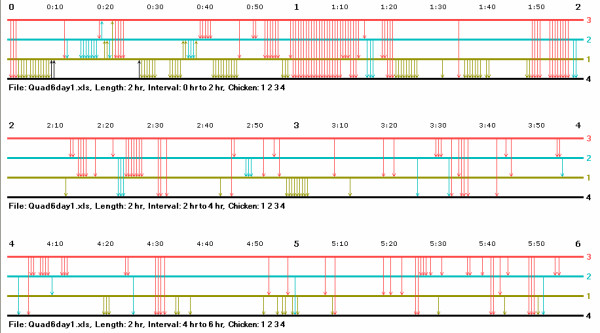
**The music notation graph for four hens on their first day of meeting**. The horizontal lines represent the hens by their ranks within their hierarchy: red for the top-ranked hen, blue for the second-ranked, green for the third-ranked, and black for the fourth-ranked. Arrows indicate aggressive acts from one hen to another, and the arrows are in the color of the initiator and go from her line to the line of the receiver. The numbers at the ends of the lines show the wing badge identification numbers of the hens, and the time in minutes and hours elapsed since the group was introduced is indicated above each block of the graph.

The simplest use of the graphical method developed here is display: providing a form in which records of interaction among group members can be directly inspected – something, as noted earlier, that is impossible to do with the usual text list form of a social interaction data set (see Table 1 for an example of a raw data excerpt). Figure 1 shows an example of the graphical method for one group of four hens forming a dominance hierarchy. A research assistant and I recorded every instance of aggressive behaviour involving physical contact among these hens from introduction through the formation of a stable, linear dominance hierarchy over the course of two days (see Methods below for more information). The figure displays the data record of all the interactions among the hens during their first day. I call this new method interaction music notation, or just music notation for short, in reference to its resemblance to the notation used in representing the notes and timing of musical compositions. In the graph, each horizontal staff line represents a different hen in the order of her eventual rank in the group hierarchy (gotten by a separate analysis of the data) starting with the top-most hen. The lines are color coded with a standard set of colors in this paper: red for the top hen, blue for the second-ranked, green for the third-ranked, and black for the bottom-ranked. These colors help make the lines visually distinct from one another and can be changed, if desired, in the program that creates the music graphs. The program allows the user to select among a variety of background colors, or select no color (white background from the screen or printer paper), depending upon color choices for the lines and arrows and personal preference. Different monitors and different printers will show slightly different versions of the colors.

**Table 1:** An excerpt from the raw data record of a group of four hens

**Time**	**Interaction**
09:31:58:0	3P4
09:32:20:3	3P4
09:32:41:7	3P4
09:33:25:6	1P4
09:33:26:8	1P4
09:33:27:2	1P4
09:33:27:5	1P4
09:34:57:7	1P4
09:34:59:3	1J4
09:35:00:5	1P4
09:35:01:3	1P4
09:35:02:3	1P4
09:35:02:7	1P4
09:35:03:1	1P4
09:35:03:4	1P4
09:35:22:2	4P1
09:35:24:0	4J1
09:40:15:4	3P1
09:40:23:9	2P1
09:46:53:3	2P1
09:46:56:6	2P1
09:46:58:9	2J1
09:47:00:7	2P1
09:47:03:7	2P1
09:47:04:4	2P1
09:47:23:7	2C1
09:47:26:0	3P2

The numbers 1 through 4 indicate the identities of the hens. Each letter indicates a different kind of aggressive contact behaviour. A "P" indicates a peck, a "J" a jump on (one hen strikes another with one or both feet), and a "C" a claw (one hen scratches another with the claws of one foot while keeping the other foot on the substrate). Each line of the table gives the time at which a behavior occurred and the identity of the hens initiating and receiving the behavior. In the music notation graphs shown in this paper all three kinds of aggressive contact behaviours are considered together as attacks. See the text for more details.

Numbers at the right-hand end of each line indicate the identification numbers from the wing badges of each hen used to identify individuals. Arrows indicate aggressive acts from one hen to another; they are drawn in the color of the initiator of an attack; and they go from the initiator's line to the line of the recipient. Attacks that coincide with the rank order of the individuals in the hierarchy – eventual or presently higher-ranking hens attacking eventual or presently lower-ranked ones – point down, while those that do not, point upward. As in regular music notation, time runs from left to right, but in the notation here time is not divided into measures, but into minutes and hours elapsed since a group was assembled and observations began. The numbers just above the top line of the notation indicate this elapsed time. The name of the file from which the data come, the length of time portrayed, the interval of time shown, and the identification numbers of the hens in the graph are indicated under each horizontal block of a music notation graph.

While we know from the earlier analysis that these hens formed a linear hierarchy and the rank of the individuals within the hierarchy, we do not know any of the behavioural details by which they formed the hierarchy or came to take their places within it. For example, how quickly was the hierarchy formed? Were all relationships in place early on or did some only form later? Did the relationships form after prolonged fights or did the hens seem to accept their positions in relationships after only a few or even no counterattacks? Were certain patterns of behaviour followed by or directed toward hens of different statuses?

The music notation graph of this group helps to reveal the behavioural patterns occurring during the formation of their hierarchy and can aid in answering some of the questions raised above. For example, in this group of hens the individual identified as number 3 (red) eventually gained dominance over all the others. She did so after being attacked only a few times by the future second- and third-ranking hens (identification numbers 2 and 1, the blue and green hens, respectively), and these attacks only occurred sporadically during about the first 20 minutes of group formation. Attacks counter to their subsequent ranks also occurred briefly between the eventual second- and third-ranking hens, as well as between the eventual third- and fourth-ranked hens. Around 40 minutes into the experiment, all attacks counter to the eventual ranks of the hens stopped. The top-ranked hen "cemented" her dominance over the others in a concerted series of attacks from about the 50-minute to the one-hour-and-twenty-minute marks, and she then continued to attack her subordinates, sometimes in short bursts of activity, over the course of the six-hour observation session. Perhaps not surprisingly, throughout much of the six-hour period of observation, the top three hens directed a large proportion of their aggressive acts to the bottom-ranked (black) hen.

A researcher can inspect a music notation graph to determine easily whether or not some animals had failed to settle their dominance relationship or whether some animals have been assigned to incorrect ranks within the hierarchy. Series of alternating up and down arrows between pairs indicate unsettled relationships, and long, stable series of up arrows from an animal ranked lower in the graph to one ranked higher would indicate that the animal shown lower in the graph actually dominated the one shown higher.

**Figure 2:**
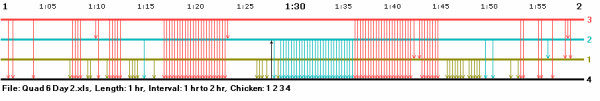
**An excerpt from a music notation graph showing a group with a high frequency of interaction**. This group had such a high frequency of interaction that the graph had to be plotted in one-hour blocks instead of the standard two-hour blocks.

In some groups the frequency of interaction may be so high that the arrows indicating the aggressive acts could not fit into the space allotted in the standard two-hour blocks provided by the program. In that case, the program gives a message to that effect, and the researcher may opt for blocks of either one-hour or 30-minute duration. On the second day of their experiment, the group just considered had stretches of time with extremely high frequencies of interaction, and the interaction record of this group had to be plotted in one-hour units. Figure 2 shows a portion of their graph for the second day – the interval from the first to the second hour – and the density of interaction, as well as its details, can be clearly seen. The interactions of another group of hens among the 14 groups observed were even more frequent and had to be plotted in 30-minute intervals.

A few things should be noted about the kind of data displayed, the graphs themselves, and the interpretation and use of the information in the graphs. First, if the data displayed with music notation are limited in some way – not all the interactions among the individuals were recorded, focal individual sampling was used (rather than observing all interactions of some type among individuals in the group), or the group was too large for all interactions of concern to be observed – the music graphs will not as clearly show the interaction processes occurring in a group as when the data are more complete and less limited. Second, music notation graphs will be most effective with groups of relatively small size, probably under 10 individuals. As group size grows, the graphs will become more visually complex, and researchers might find it more difficult to perceive patterns of interaction. However, this may be a moot point since it is extremely difficult to collect high quality data showing all relevant interactions among larger groups of animals. Also as Krebs and Davis [3] note, larger groups may have more trouble forming linear hierarchies, and in some species, these larger groups will split into subgroups. These subgroups could of course be more easily handled in music notation graphs (see below for the portrayal of subgroups with music notation). I elaborate on group size issues in the Discussion. Third, researchers with certain kinds of colour blindness might find it difficult to distinguish between some of the colours used for the hens in the graphs displayed here. Potential solutions involve restricted colour choice or use of gradations along a gray scale; see the Discussion for more details. Fourth, as is the case with any other visualization tool, music notation can help a researcher recognize patterns, regularities or differences, in one data record or between data records, but how to use that information to develop hypotheses, carry out statistical test, design new experiments, formulate new models, etc. is beyond the scope of music notation itself.

### Comparison of interaction records: groups of real animals and real animals versus computer simulations of hierarchy formation

The music notation graph for the group of hens considered in Figure 1 shows a few attacks back and forth among the eventual top three animals and between the eventual third- and fourth-ranked hens before relationships and ranks become stable. But is this pattern typical? Are initial aggressive interactions counter to eventual ranks common or rare on the way to hierarchy formation? What about high frequencies of interaction for the first two hours of hierarchy formation and high frequencies of attacking the bottom animal by the top three? Just how similar, or different, are the interaction records of different groups establishing hierarchies? And in a related manner, how similar are the interaction records of groups of real animals forming hierarchies to those of computer simulations attempting to model hierarchy formation? The examples below illustrate how music notation graphs can be used to help answer questions such as these.

#### Comparing interaction tecords in groups of real animals

**Figure 3:**
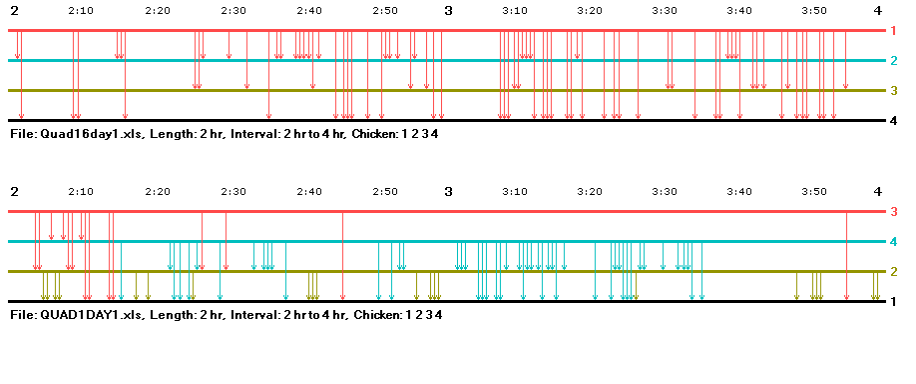
**A comparison of interaction records in two group of hens**. This figure illustrates the comparison feature of the music notation program showing the interaction records in two groups of hens interleaved in two-hour blocks.

In order to help researchers develop their intuition concerning similarities and differences in interaction records and to develop testable hypotheses concerning interaction patterns across groups, the music notation program has a feature that facilitates the simultaneous comparison of the interaction records of two groups at a time. Figure 3 illustrates an example of this feature showing a portion of the records of two groups interleaved in two-hour blocks (the individuals in a group always had the same wing badge numbers – 1, 2, 3, and 4 – but they were different individuals). Two kinds of differences in the hierarchy formation process of these groups can be easily seen. In the first group, the top-ranked hen initiates all of the aggressive acts, there are no counterattacks, and the frequency of aggressive acts is fairly high during the observation period. (The dominance relationships among the lower-ranked hens in this group filled in on the second day of observation.) In the second group, the interaction rate of the top-ranked hen is relatively low, but the lower-ranking hens interact with one another (perhaps because of the low rate of interaction of the top hen), and all the pairs of hens making up the group had dominance interactions during the first day of observation. The interleaving of the group records helps makes the comparison between groups easier in that the researcher can quickly and easily scan back and forth from the graph of one group to that of the other.

Having used music notation to see these two very different "styles" of hierarchy formation, a researcher might ask whether there were yet other styles, or whether a certain number of styles were common, within one species or across various species. Along these lines, two fundamental questions can be asked about different styles of hierarchy formation: Do some common behavioral processes underlie all these different styles? In spite of appearing so different, how is it that the interaction records in both groups (as well as in all the other groups in this study) lead, as they do in the end, to linear hierarchies? Comparing a series of groups using this feature of the music notation software could be of great help in working out answers to these questions.

#### Comparing interaction records in groups of real animals with those generated by computer simulations

In the last twenty years or so, researchers have developed a variety of models using winner and/or loser effects and sometimes bystander effects, usually implemented through computer simulation, in attempts to account for the development of linear dominance hierarchies (e.g., see [4-7]). These models can generate interaction records that do indeed lead to linear hierarchies when animals are assumed not to identify each other as distinct individuals, not to remember the outcomes of past contests, and when certain levels of parameters concerning the influence of winning and losing earlier contests are chosen. However, the interaction records produced by these models have never been checked against the interaction records of real animals forming dominance hierarchies. As a result, we do not know whether or not these simulated interaction records resemble those in real animals, and, consequently, whether or not the computer simulations are generating linear hierarchies through the same sorts of interaction processes that real animals actually use.

**Figure 4:**
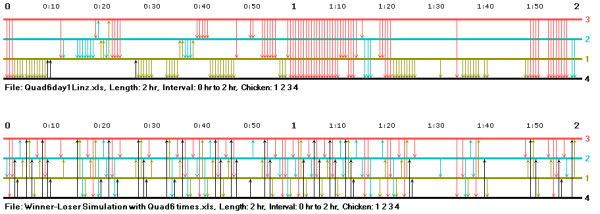
**A comparison of interaction in real hens versus a computer simulation of hierarchy formation**. The first two hours of interaction during in dominance hierarchy formation in a group of real hens is compared to simulated data on hierarchy formation using a modified form of Hemelrijk's [5] model based on winner and loser effects. The acts in the simulated data records are given the same timing as the respective acts in the real data. See the text for further details.

The comparison feature of the music notation program facilitates the easy juxtaposition of real and simulated interaction records, and thus can help researchers compare the ways in which real animals interact with those suggested by computer simulations and mathematical models. After comparing a number of real and simulated records, researchers could then go on to ask questions about similarities and differences between the two types of records and to develop more formal goodness of fit tests. For example, the various real records presented so far in this paper show some, but relatively few attacks counter to eventual or current rank orderings in groups: Is this also a feature found in the simulation models? In other words, the hens in the graphs formed their hierarchies in what might be termed a relatively "efficient" manner – but do computer models show comparable levels of efficiency? To illustrate, Figure 4 gives a comparison between the first two hours of interaction in the real group of hens shown previously in Figure 1 and a record of simulated interaction based upon a modified version of Hemelrijk's [5] very thoughtful model for the formation of dominance hierarchies. In order to make the comparison between interaction in the real group and the simulated one easier to see, I have given each successive act in the simulated record the time of the comparable act in the real record. In other words, if the first act in the real record occurred at 2 minutes after observation began, the first act in the simulated record was assumed to occur 2 minutes after observation began, and so on.

An examination of the two interaction records reveals that they are remarkably different. As indicated above, the real animals form their hierarchy "efficiently," with relatively few attacks back and forth between future dominants and subordinates, while hierarchy formation in the simulated animals is extremely "inefficient" with repeated attacks back and forth among them. In addition, the higher-ranking real hens often attacked their subordinates quite a few times in a row while the simulated animals did not. Although additional research would have to be done, using music notation graphs to make this brief comparison suggests that the real hens may not use the same basic interaction processes in forming their hierarchies as hypothesized in this computer simulation.

As noted in the previous section, prescribing statistical tests, new models, new experiments, etc. to evaluate patterns or differences in patterns found with music notation graphs is beyond the scope of the visualization methods themselves. However, the present comparison suggests that the real and simulated records might be compared statistically using such things as the average number of aggressive exchanges (attacks and counter attacks) that pairs of animals have before they establish stable dominance relationships or the average total number of attacks among all pairs in a group before a stable hierarchy is reached. In addition, the present comparison also suggests that the present simulation model might be changed to incorporate some full or partial memory of the results of past encounters and to have interactions arranged in bouts in which an individual might attack one or several others in a row before another individual began a series of attacks.

### Prospecting for small-scale interaction patterns

Music notation graphs can aid researchers in searching for various small-scale patterns of interaction in groups as well as in checking for the presence of interaction patterns hypothesized to exist in theoretical models, but not yet examined in groups. Below I give an example of each type of activity: first, one concerning a pattern not often studied outside of primate groups, and then some patterns that are crucial to the computer simulations of linear hierarchy formation just discussed.

**Figure 5:**
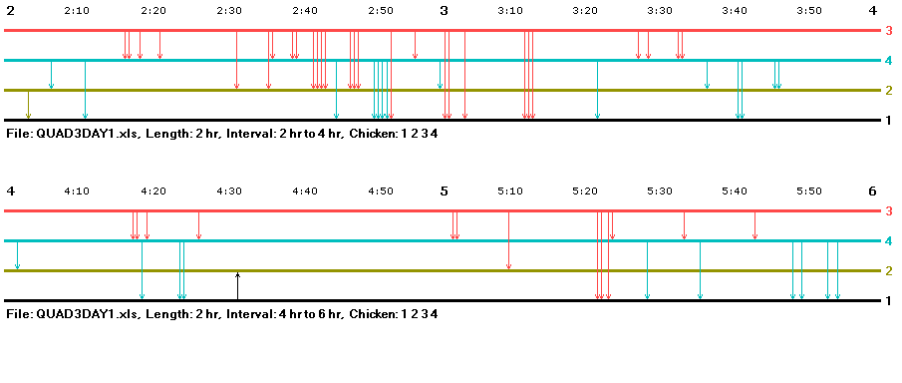
**An excerpt of a music notation graph used to prospect for "policing" behavior**. In this graph single attacks or short series of attacks by the second-ranking hen on her subordinates are usually followed within ten minutes by one or more attacks of the highest-ranking hen on the second-ranking one.

In primate groups, researchers have noted a behavioural pattern in which higher-ranking animals respond to the attacks of middle-ranking animals on lower-ranking ones by attacking the middle-ranking animals. This is sometimes referred to as "policing", although this term also refers more broadly to interventions that help terminate conflict and are equally directed to both participants, rather than favouring one animal of a pair. (e.g., see [8-11]). (In the social insect literature "policing" has a different meaning indicating worker interference in the reproduction of males by other workers). Are attacks of higher-ranking hens on middle-ranking hens that have just attacked their lower-ranking subordinates a behavioural pattern that might have occurred in the groups being examined here? Would it occur commonly enough to be considered as an actual process of interaction rather than just an occasional and, therefore, probably chance occurrence? Consider, for example, Figure 5, which shows the last four hours of interaction in a group of hens on the first day of observation and just after the dominance relationships in this group have filled in to form a linear hierarchy. Inspection of this graph shows that in the case of single attacks or short bursts of attacks by the blue, second-ranking hen on either of its subordinates (green and black), the red or top-ranking hen usually attacks her (blue) within about 10 minutes. This happens in some seven out of nine instances of attacks by the blue hen – not counting the burst of attacks just before the observation session ended. Is this a significantly common interaction pattern across groups? If inspection of the music graphs of other groups suggested that it might be, then this hypothesis could be tested by more formal analysis of the entire set of interaction records for the hens.

**Figure 6:**
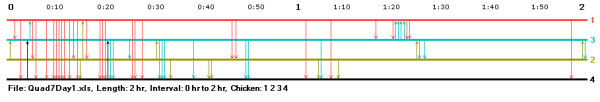
**An excerpt from a graph raising questions about winner, loser, and bystander effects**. This excerpt from a music notation graph of four hens illustrates the difficulties of clearly and unambiguously defining winner, loser, and bystander effects and which acts might represent particular effects during the formation of dominance hierarchies.

Above, I indicated that the music notation program could be used to compare the interaction records of real groups forming hierarchies versus those generated by computer simulations, and I noted that most of the current computer models rely on winner and/or loser effects and that some also incorporate bystander effects [4-7]. While experimental work shows that these effects occur in isolated pairs of animals, as far as I am aware, only one study has investigated whether or not any of these effects might occur in larger groups forming hierarchies, as the models assume. This study investigated the loser effect, along with several other effects found in isolated pairs, and found that it did not occur in larger groups [1]. However, that study used only one species – of fish – and it did not examine this effect during the course of hierarchy formation, but only on the final outcome of dominance relationships in groups. Perhaps some small-scale interaction patterns that we could associate with winner or loser effects actually do occur during hierarchy formation. Inspection of the music notation graphs of various groups forming hierarchies could help make this clear. Figure 6 shows an example of the first two hours of observation in a group with a fairly complex record of interaction, and one raising questions that need to be considered in associating small-scale interaction patterns in groups with winner, loser, and bystander effects. For example, after the very first attack in this group, (which, by definition, cannot show either a winner, loser, or bystander effect), the next two attacks seem consistent with winner, loser, and bystander effects: (1) red attacks blue following green's attack on blue and then (2) red goes on to attack black. The first action, red's attack on blue following green's attack on blue, could be considered as either coming from a loser effect – losers (like blue) have an increased probability of losing their next encounter – or a bystander-effect – an animal observed to be attacked (like blue) is more likely to be attacked by another animal (like red observing the attack). The second action, red's attack on black immediately following red's attack on blue, could be judged as either coming from a winner effect – a winner (like red) has an increased probability of winning its next encounter – or from a bystander effect – an animal observed to have attacked one animal (like red) is more likely to be submitted to by an animal observing this attack (like black). But the next three attacks are more problematic: (3) black attacks blue, (4) blue attacks red and (5) red attacks green. All three of these attacks go against the loser effect (and some against the winner and bystander effects as well) in that an animal just attacked goes on to attack another animal. More generally, this example helps to show that attempts to define, much less to verify, the presence of winner, loser, and bystander effects in the interaction records of groups forming hierarchies is not as straightforward as it is in the usual experiments involving just two animals [1]. For example, should these effects be defined in terms of the most recent experience of the initiator or recipient of an action, or should some new definitions of these effects be developed that encompass the complexities of group interaction? It might often be difficult to code attacks as clearly showing only one of the possible effects, and some specific attacks could show all of the possible effects or none of the possible effects, depending upon how the effects were defined. For example, if A has attacked B and then A goes on to attack C, who has just attacked D and who has been attacked earlier by B, is A's attack on C an indication of the winner effect (considering only A's previous attack on B), an indication of the bystander effect (considering A's possible observation of B's previous attack on C), an indication of no effect (both A and C are equivalent in that both have been previous winners in their most recent attacks), an instance of the loser effect (considering only B's previous attack on C), or an instance of a violation of the winner effect (considering only C's attack on D)? Using music notation graphs can help researchers to gain insight into whether or not interactions showing winner, loser, and bystander effects are components of the hierarchy formation process, but only after researchers develop new definitions of how these effects might manifest themselves in group contexts. More generally, this example points out the potential problems of attempting to generalize ideas about interaction processes in isolated pairs to hypotheses about interaction processes in group contexts. The use of music notation graphs can help illustrate this problem and aid researchers in discovering the forms of interaction processes that actually do occur in larger groups.

### Examination of interaction records in subgroups

Consider a larger group within which subgroups of two or three animals are contesting with each other for dominance: animals A and B might periodically attack and counterattack one another at the same time as B, C, and D are also contesting with one another to work out their relationships. In a case like this, the aggressive interactions involved in the "power struggles" in the separate subgroups would probably be interspersed in time rather than be clearly sorted out in separate and distinct bouts, and, as a result, the overall record of interaction in the whole group might appear complex and hard to make sense of. As an aid in sorting out situations of this sort, the music notation program has a feature that allows a researcher to plot interactions in various subgroups concurrently with interaction in the whole group.

**Figure 7:**
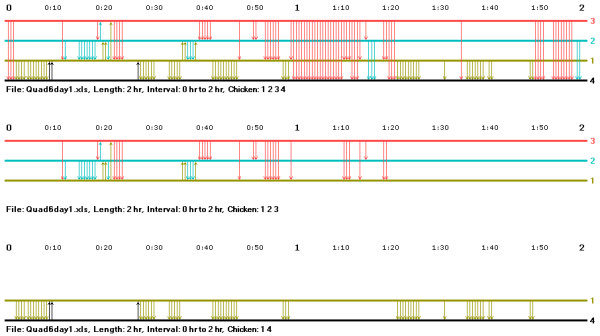
**A comparison of dominance interactions in a whole group with those in two component subgroups**. This figure shows simultaneous records of interaction during dominance hierarchy formation in a group of four hens and in two different component subgroups. This feature can be used to search for the presence of such behavioural patterns as "power struggles" in the subgroups making up a larger group.

Figure 7 illustrates the use of this feature. This figure shows the first two hours of interaction in the group of hens portrayed in Figure 1, but now broken down into two separate subgroups – the top three hens (red, blue, and green) and the bottom two (green and black) which have simultaneous, but short, power struggles in the initial stages of hierarchy formation. At the end of the second hour of observation the relationships begin to consolidate, and in the last two hours of interaction in the group (not shown in the graph), there are no counterattacks and a clear linear hierarchy is evident. Without this feature of breaking out the interaction records in the two subgroups, the record for the whole group appears much more intricate and shows much less organization.

Are such, perhaps brief, power struggles within subgroups a common feature of hierarchy formation in some species and less common in others? Do the results of interactions in one subgroup propel animals to take actions against animals in another subgroup? Or more generally, is the idea of processes of interaction in subgroups more helpful in understanding the establishment of hierarchies in some species than in others? As noted above, inspection of music graphs shown here for the hens suggested that they form their hierarchies without prolonged bouts of attacks and counterattacks between pairs. However, some species, perhaps of primates, may not share this feature and breaking down a larger group's interaction record into music graphs for sub-groups could be especially helpful for them. Inspection of music notation graphs for a variety of species would be of considerable help in developing and exploring questions of this sort.

## Discussion

For over 200 years researchers have been developing graphical techniques that allow us to use our capacities for visual pattern recognition to see regularities in quantitative data that would otherwise be difficult to discern [12-14]. This paper provides an example of how we might use those same capacities with new graphical techniques such as music notation graphs to understand better social interaction in human and animal groups. I suggest that music notation graphs can be of particular help in a variety of fields interested in social interaction in humans, animals, and machines such as behavioural ecology, behavioural economics, social organization in animals, development of social networks in humans, human conversational analysis, and the coordination of actions in social robots.

In this paper, I have shown how the basic music notation concept can be used to display extensive, detailed records of interaction in an easily seen and understood format; to facilitate the comparison of processes of interaction in different groups of real animals and in real animals versus those generated by computer simulations; to discover the occurrence and context of small-scale sequences of interaction during the formation of groups; and to break down the interaction records of a larger group into simultaneous records of interaction in different subgroups. While I have illustrated these different possibilities using the example of hens forming dominance hierarchies, I have suggested that music notation could be used as a general method for visualizing many kinds of social interaction in groups of animals and humans. Let me raise several considerations that are important in doing this: limitations on the number of group members that can be displayed, showing different kinds of behavioral acts in the same music notation graph, indicating behaviours with durations, and layering other sorts of data with music notation displays, showing simultaneous interactions, and facilitating the use of music notation displays by colour-blind researchers.

First, concerning group size, music notation techniques are probably best suited for groups with relatively few members. I have experimented with hypothetical data on hierarchy formation, and here I would think that interactions among seven to ten individuals are the maximum that can be shown clearly with music notation graphs. With larger numbers of individuals it becomes increasingly difficult to pick colors that allow a user to clearly distinguish among the lines and arrows representing the individuals. However, choosing a color background for the graph, such as a medium or dark blue, rather than just using white as I have here, makes it easier to chose a larger set of distinct colors for individuals. Showing interaction among a larger number of individuals becomes especially difficult when the interaction is not well-ordered, for example, if there are many attacks and counter-attacks between individuals. But if the interactions are well-ordered or relatively infrequent, it might be possible to make good use of music notation graphs at the upper end of this range, or perhaps even extend it a bit.

Second, in displaying different kinds or intensities of behavioural acts, the colours of arrows and the shapes of both arrowheads and arrow shafts might be altered. For example, in order to show differing intensities of interaction, say, from threat displays to physical aggression in two-animals contests, a basic color could be chosen for each animal with lighter shades indicating threat behaviors and darker shades indicating escalating physical contacts. In the case of completely different kinds of behaviors, the heads or shafts of the arrows could be altered. For example, I could have used modified heads to distinguish different kinds of aggressive contacts in the chickens, say, a regular arrowhead (the kind shown in the graphs) for a peck; a small, closed circle for one chicken scratching another; and a small, open square for one chicken striking another with its foot. In other situations arrow shafts might be modified, for example, in small human groups, a dotted shaft might signify an interruption, a wavy shaft a question, and a standard shaft, like the one used here, a statement. In portraying, for example, two-person athletic contests, modified heads or shafts might be used to indicate different sorts of punches or thrusts in, say, boxing or fencing matches. Punches or thrusts that connected with the opponent could be signified by arrows that touched the opponent's line, while those that did not could stop short of this line. Using different colors and modified shafts and heads would impose greater cognitive loads on users of music notation graphs than the sorts of graphs shown here, and these loads would have to be considered and kept as small as possible in any modifications and extensions of music notation.

Third, in the case of behaviors having duration, as opposed to the more or less instantaneous ones considered here, arrows would not be satisfactory. For behaviours with duration, a possible modification might be to use line segments or bars in the color of the initiator, corresponding to the duration of the behaviour, and laid down on the line of the receiver. For example, such a modification could be used to show the length of time that two or more primates took in grooming each other.

Fourth, in the case of layering other types of behavioral information about individuals with music notation graphs, it might be very helpful to know such things as heart rate, blood pressure, respiration rate, vocalizations, etc. that occurred at specific times in bouts of interaction. For example, having a music notation graph of interaction combined with a record showing things of this type, on the same time scale, could help researchers pinpoint the particular behavioral contexts in which specific vocalizations were used in primates or birds, or what kinds of interactions led to increases or drops in heart or respiration rates.

Fifth, in small groups of individuals, such as those portrayed here, only one pair of animals usually interacts at a time. However, in larger groups, but still within the size limit appropriate to music notation, two or more pairs might interact simultaneously. To present these simultaneous interactions, the standard display for a group shown here could be modified to present two music graphs for the group for the same time interval – one over the other – as in the method for showing the graph of a group along with those of component subgroups (Figure 7). The top graph could present the bulk of the interaction in the group while the bottom graph could display those acts, probably few in number, which occurred simultaneously with certain of the acts shown in the upper graph. For example, if at some point, A attacked B at the same instant as C attacked D, the interaction between, say, A and B could be presented in the upper graph at the time it occurred (along with all the other non-simultaneous acts that occurred in the group), and the simultaneous interaction between C and D could be presented on the lower graph directly below the A-B interaction.

Sixth, in order to help facilitate the use of music notation by colour-blind researchers, I can think of two possible approaches. One would be to alter the colours for the individuals with a particular kind of colour-blindness. For example, for those with red-green colour-blindness, one or both of those colours could be avoided, and one or more other colours that could be distinguished could be substituted. A second approach would be to apportion the lines and arrows along a grey scale: this should work for all the various varieties of colour-blindness. Here the lines might go from very light grey, through mid-greys to black. However, the use of a grey scale would probably restrict the numbers of animals that could be displayed to less than those possible using colours, since it might be difficult to distinguish easily a large number of different grey tones.

## Conclusion

While social interaction is an important concern in many areas of study within animal behaviour and the social sciences, there are relatively few graphical techniques that display raw interaction records (but see, for example, [10,15] for techniques for visualizing interaction in social networks). In this paper I have introduced a new technique that allows researchers to visualize interaction in groups of humans and animals in an easily grasped way. This technique can help researcher discern patterns in interaction not easily seen through other means, to compare interaction records in real and simulated groups, to prospect for various kinds of small-scale interaction sequences, and to break down interaction records in a larger group to those occurring in several component subgroups. I have, by way of illustrating the power of music notation visualizations, raised a number of basic questions about the dynamics of the hierarchy formation process, including ones about the frequency of attacks counter to the eventual or present ranks of animals in a hierarchy; the rate of interaction at various stages in hierarchy formation; similarity and dissimilarity of interaction records across different real groups and between real groups and simulations of hierarchy formation; possible influences of winner, loser, and bystander effects on the outcomes of contests; the presence of small-scale sequences involving "policing" behaviour; and processes of interaction taking place in subgroups during hierarchy formation. The use of music notation graphs to raise questions such as these can help researchers in explaining behavioural processes, deciding upon appropriate statistical analyses of interaction data, formulating new models of social interaction, and designing new studies of group processes.

## Methods

### Experimental animals and data collection

A detailed description of the experimental methods can be found in Chase [16]. Briefly, 14 groups of four 3-year old white Leghorn hens each were assembled from a pool of 21 hens using a balanced, incomplete block design [17]. This design gave a procedure for assembling smaller groups from a larger pool such that no two individuals met more than once, all individuals were in the same number of groups, and all individuals in the experiment took part in the same number of groups.

Two observers taking alternate 1.5 hour shifts and working from behind a blind recorded the behavior of the chickens for six hours a day for two successive days for a total of 12 hours. The hens were tested in a 152 × 102 × 81 cm. cage with food and water available *ad libitum*. When the hens were not being observed, they were separated by opaque partitions. The observers recorded all aggressive interactions among the hens involving physical contact. In 168 hours of total observation for the 14 groups of four, they recorded 7402 acts for an average of 44.1 acts per hour or 528.7 acts per group.

This research followed internationally recognized guidelines. The research protocol was examined and approved by the Chief Veterinarian and Director of the Division of Laboratory Animal Resources at Stony Brook University according to the standard university policy at that time (1979).

### Algorithm for the graphical display of data

The present program for displaying the aggressive interactions among the hens is written in Visual Basic, and it can plot interactions in groups of two to four individuals. The program accepts data from EXCEL files with column one of each line indicating the time at which an interaction occurred and column two indicating the particular interaction that took place at that time (see Table 1). The program requires, as input, the individual identifications of the animals and their ranks within the resulting hierarchy. The program supplies a color chart so that a researcher can pick colors clearly distinguishing the lines and arrows representing each individual as well as the background color for a graph (see above). The program writes the graphical output as an HTML file making for ease of sharing files with other researchers, if desired, and to increase the portability of the files. A new software package for visualizing a wide variety of different types of interaction in humans and animals is in preparation. (The present program is available from the author upon request.)

## Competing interests

The author(s) declare that they have no competing interests.
